# Man and His Enemies

**Published:** 1889-04-13

**Authors:** 


### The Doctor's Pulpit.

### MAN AND HIS ENEMIES.

### Breaking to Harness.

All the domestic animals have to learn to live by rule. Man has to learn to live both by rule and reason. Animals whose reasoning faculties are poorly developed are taught to live by rule with great difficulty, and are apt to break away from authority on small provocation. If, in addition to feeble reasoning powers, there be in certain classes of creatures strong passions, such as hunger, rage, and so on, it is often impossible to teach or compel them to live by rule at all. It is remarkable what resemblances may be discovered between individual human beings and different classes of mammalian animals. Each natural order of mammals has its own peculiar characteristics, and those not merely of form, feature, and colour, but also of disposition and temper. There is no difficulty, for example, in realising clearly the difference in temper between a lion and a sheep, or between a horse and a bear. Very similar contrasts may be observed between different human beings, and to a less extent between different races. It seems as if man had inherited the capacities and potentialities of all the creatures lower in the scale of development than himself, as if in passing through the progressive stages of his being he had finally emerged as the leading type and sublimated essence of the whole animal world.

In considering the young human being at the stage of life we have now reached, the stage of "breaking to harness," it is of the most fundamental importance rightly to understand the actual character and contents of the mind and temper of each separate individual. The educator of youth—whether schoolmaster or superintendent worker—if he be of a philosophical and practical temperament, as he should be, may learn invaluable lessons from the "breaker" of young horses, the tamer of lions, the trainer of the different kinds of dogs, the driver of elephants, and other persons of the same class. All these animals have, so to speak, to be "broken to harness" at certain periods of life; and it is well understood that those periods are both critical and dangerous. This is emphatically the case with boys and girls at the "school" age. Their headstrong passions, their inexperience, and their general recklessness, make that stage of their development one of the most dangerous of all.

It is an infinite pity for schoolboys and schoolgirls that so many parents and teachers have so little sense and such poor training powers. What is the reason why the lower animals are so difficult to tame and teach? Because they have so little intellect. When a trainer meets with an animal possessed of more than average mental capacity he congratulates himself that his task is practically accomplished. But if animals are difficult to train in proportion to their deficiency in intellect, ought not human beings to be correspondingly easy because of their possession of such marked intellectual powers? Compared with the lower animals, every sane human being is a creature of high intellectual capacity, and should, in proper hands, be quite easy to train and "break to harness."

But the intellectual faculty is not the only advantage which the young human being, regarded as an animal to be trained, possesses over the other mammals. There is, in addition, a moral sense of a more or less marked character; and that is a lever, in the bands of those who know how to use it, which will, like Archimedes' lever, move the whole world of mind when its right fulcrum is found. If the active generation of parents and tutors of youth were all as competent for their duties as an ordinary lion-tamer, or trainer of circus horses, a revolution would be produced in human affairs in the course of two or three decades such as has not resulted from what may be called the "natural development" of tens of centuries. We look at boys and girls as bodies, as minds, as souls. So looked at, we can see that parents and teachers can affect them injuriously or advantageously, both in body, in mind and in soul. They can, in short, be friends or enemies. Of course it need not be supposed for a moment that parents and teachers will be enemies by choice. But they may be enemies by ignorance, enemies by carelessness, enemies by perversity, enemies by sheer stupidity.

A schoolboy may be said to be his own enemy, in that he recklessly exposes life and limb to every imaginable risk and danger. Every boy in the same community may be looked upon as every other boy's enemy, because, on the smallest provocation, the hot blood of youth is inflamed, and two human creatures will fight each other with all the savage ferocity of wild beasts or gamecocks; or with all the ridiculous feebleness of infuriated lap dogs. But these are mere trifles compared with the permanent injury which may be done to the future man by unwholesome material environment or injurious mental and moral conditions. The oak tree depends for its development upon soil and climate. These two are the principal circumstances which determine its value as an oak tree. Similarly, boys and girls depend upon their natural constitutions and their food and housing, on the one hand, and on the influence exercised upon them by the intellectual and moral potency of parents, relatives, and teachers, on the other. The former may be called the soil in which they grow; the latter the climate under which they live.

With man, reason is before everything; reason, which includes duty. Even the physical conditions under which all human beings grow up are, to a large extent, the product and fruit of reason. That is the not the case with the wild animals. They find themselves in a certain environment, and there they must remain and adapt their ways to their circumstances. But with man it is quite otherwise. In every stage of civilisation he can, in a great measure, make his own environment, both physical and intellectual. He is, so to speak, master of his own soil and climate. But that is surely a great power which he possesses—a striking advantage over all other animals. Of course it may be argued that, as a matter of fact, man does not make his own environment, but grows up amid the surroundings in which he has been born, and is modified by them, and modifies them in turn, exactly as other animals do. True! But the position here affirmed is, not that man always does modify and more or less completely make his own environment, but that, being possessed of reason and free will, he has the intelligence and the physical power which fit him to do so. He can do it as an individual if he will; he can do it in co-operation with his fellows if he and they choose to combine. That he does not do it, except in a poor and unworthy degree, is his fault much more than it is his misfortune.

It is one of the most obvious of sociological facts that the adult world that now is creates and maintains conditions of a physical, intellectual, and moral kind which are often dangerous and sometimes destructive to the coming generation. Every smoky town, every foul river, every poisonous drain, every pestilential fever house, every drunkard, every thief, every fallen woman, every insincere and impure-hearted man, every idler, every frivolous person, every fool, conspires with every other creature of the same class to create an environment in which the young of the human species must physically and intellectually breathe and live. This is the baldest possible statement of a scientific sociological fact. A pure atmosphere and a congenial soil are the primary and indispensable conditions of all healthy life of every kind. How can our boys and girls be either physically or intellectually sound and wholesome in the environment in which they are placed by our action and the action of our forefathers? What a vast amount of self-preserving antiseptic substance must be inherent in human bodies and minds when so many of them can preserve themselves from physical and intellectual death and putridity under such unfavourable circumstances as these! Talk of human nature being exhausted and worn out! It is only just beginning to live! What we need in these days is the marriage of a true and intelligent science with a reasonable and universal religion; in other words, a combination of the highest knowledge with the deepest recognition of duty. Then we shall have an atmosphere and a soil—a complete environment—in which perfect physical, intellectual, and moral health will be not only possible but inevitable. That is the end towards which all men who have insight and conscience should steadfastly aim.

